# Timeliness of reporting process in the national routine health information system: The case of 19-year experience of Field Health Services Information System in Palawan, the Philippines

**DOI:** 10.1371/journal.pone.0264681

**Published:** 2022-02-25

**Authors:** Shinsuke Murai, Ray Justin C. Ventura, Julita T. Gaite

**Affiliations:** 1 Bureau of International Health Cooperation, National Center for Global Health and Medicine, Shinjuku, Tokyo, Japan; 2 Department of Health, Bureau of Local Health Systems Development, Manila, Philippines; 3 Monitoring Planning Research Epidemiology and Surveillance Division, Provincial Health Office of Palawan, Puerto Princesa, Palawan, Philippines; University of Hong Kong, HONG KONG

## Abstract

**Introduction:**

Routine health information system (RHIS) has been repeatedly updated to provide quality information. However, its timeliness has rarely been tracked. This study investigated the reporting status and the timeliness of quarterly reports of the national RHIS in the Philippines, based on its 19 years-operation in Palawan.

**Methods:**

We analyzed the timeliness of 94.7% (1568/1656) of the quarterly reports that we obtained the date of receipt submitted by 22 health centers in Palawan from 1996 to 2014. The RHIS update in 2008 increased the number of reporting items and extended the submission due date since 2009 while computerized 15 health centers since 2011. We performed Fisher’s exact test to examine changing the proportion of timely reports and multiple comparisons with permutation tests for changing the mean of the quarterly median lead times, median delays and interquartile ranges (IQR), for four periods of different operational requirements in the RHIS.

**Results:**

The update increased the timely reports from 6.7% (70/1045) to 22.4% (117/523) (p<0.001). The delay remained stable from 14.2 days to 16.1 days (p = 0.654). However, the IQR widened 2.31 times (p = 0.004) compared to 15.7. Despite the increased burden, the continued manual data processing decreased the delay by 7.1 days (p = 0.023) and remained the IQR stable at 1.19 times (p = 0.670), while 15 health centers were computerized, it increased the delay by 6.4 days (p = 0.037) and widened the IQR by 2.87 times (p = 0).

**Conclusions:**

More attention must be paid to controlling the timeliness of RHIS when we introduce new interventions and perform daily management. Extending the due date increased timely reports. However, introducing unfamiliar tasks increased delay and uncertainty in timeliness. In a low-resource setting, an effective intervention needs to consider modest operating procedure changes that extend the existing routines to which the staff in charge has already accustomed.

## Introduction

The management of local health systems, especially in low-resource settings, requires relevant indicators and quality data on health system operations [1]. Since routine health information system (RHIS) provides empirical data that allow members of the organization to track their progress [2], RHIS has the potential for the organization to understand the current state of the health system and update their understanding by accumulating additional operational experience. In this way, learning is a feedback process [3]. Better feedback is expected to facilitate organizational learning and thus improve the management of the local health system. However, delayed reporting in RHIS have been repeatedly noted in several countries [4, 5]. This situation is particularly critical when local health systems rely on RHIS for most available data.

In the Philippines, the Field Health Services Information System (FHSIS) is the national facility-based RHIS. Its original system had been in operation by the Department of Health (DOH) since 1960, with minor updates until 1984 [6]. The first major update of the FHSIS was in 1989 in collaboration with the World Health Organization Regional Office for the Western Pacific. The second major update was in 1996 to accommodate the use of the FHSIS under the devolution [7, 8]. It was hoped that the update would solve incomplete and delayed reporting problems in the earlier FHSIS. However, similar problems persisted [9]. The third major update took place in 2008 and was implemented in January 2009. The Center for Health Developments (Regional Health Offices) introduced the electronic reporting system called the eFHSIS in January 2011. Like in the FHSIS in the Philippines, RHISs in other countries are repeatedly being updated. However, their reporting status and the timeliness of report submissions have been rarely tracked over time. This situation hinders identifying opportunities for improvement in both the design and operations of RHIS.

This study aimed to investigate the reporting status and the timeliness of quarterly reports of the FHSIS in the Philippines, based on its 19 years-operation in Palawan province.

## Materials and methods

### Ethical consideration

This study did not collect samples from humans; however, the data set reflects the operation of the local government of the Philippines and has the potential risk of identifying municipalities with failures in terms of timeliness. We think such failures are sensitive if interpreted beyond the framework of this study. A researcher who would like to access this data set may directly contact the Provincial Health Officer of the Provincial Health Office of Palawan (e-mail: palawanpho@yahoo.com.ph).

### Data collection

The Provincial Health Office of Palawan had 22 health centers in its catchment area. The frequency of health service reports submission from the health centers to the Provincial Health Office has been standardized quarterly since 1996. From 1996 to 2004, we reviewed 1672 quarterly reports for their reporting status and receipt dates.

We confirmed the report submission by actual reports stored in the Provincial Health Office. We obtained the earliest receipt dates from the receiving logbook of the FHSIS coordinator. It was a well-established routine to chronologically record the receipt dates on the receiving logbook. The back page of the report folders and the stamps on the reports were complementary sources of the receipt dates, which we used only when the receipt dates were unknown on the receiving logbook. The earliest receipt dates on the two complementary sources were not always consistent. For example, the date sometimes reflected the most recent receipt date when the report was returned for further correction and resubmitted to the FHSIS coordinator.

We retrospectively collected the data in June 2015 for the reports from 1996 to 2008. We prospectively collected the data annually every June of the succeeding year for the reports from 2009 to 2014. Although one additional health center has started to join the FHSIS operation since 2013, we excluded those samples because no corresponding samples before 2013 were available.

### Measures and analysis

We analyzed the timeliness of 94.7% (1568/1656) of the quarterly reports that we obtained the receipt date submitted by 22 health centers in Palawan from 1996 to 2014.

#### Differences in operational requirements before and after the guideline update

The reporting timeliness is built by the processes such as counting the number of eligible cases, data encoding, data consolidation, and report submission. Table 1 shows the differences of operational requirements before and after the update in 2008. For data processing, the FHSIS guideline required manual data processing from 1996 to 2010 and computerized data processing from 2011, and it extended the submission due date by two weeks. For counting, encoding and consolidating processes, the update added 353 reporting items and 20 columns for analysis. Although the counting process remained manual, computerization required a new task to encode the counting results into a computer program. Computerization was expected to improve timeliness by automatic consolidation and submitting reports online. As a result, 15 health centers used computerized data processing (Type 1), and seven health centers continued manual data processing (Type 2) from 2011 to 2014.

**Table 1:** Characteristics of FHSIS before and after the update.

Updates	Period (Year)	Due Date	Frequency	Processing	Items	Columns
Before	1996–2008	Every first week	Quarterly	Manual	78	None
After	2009–2014	Every third week	Quarterly	Manual (2009–2010)	431	20
				Computerized (2011–2014)		

#### The number of quarterly reports received within the due date

We counted the quarterly numbers of reports received within the due date. The FHSIS guideline defined the due date before the update as the seventh day [8] and the due date after the update as the 21st day in the first month of the succeeding quarter [10]. We performed Fisher’s exact test to examine the improvement of the proportions of timely reports according to the different operational requirements brought by the update.

#### Definition of timeliness

To visualize the quarterly dynamics of the timeliness in the FHSIS reporting process, we calculated the median lead times (days), median delay (days) and interquartile range (IQR) among 22 health centers for each of 76 quarters. We applied the definition of sample quantiles by the true rank probability [11]. Lead time, defined as the absolute number of days to submit a report to the Provincial Health Office, indicates the performance of timeliness. The starting point for the lead time was the last day of the last month of the previous quarter because cases seen on the last day of the previous quarter were supposed to be included in the following quarterly report. Delay, defined as the subjective timeliness based on the due dates, indicates the quality of timeliness. The starting point of the delay before the update was the seventh day [8], and after the update was the 21st day [10]. We followed this definition strictly to simplify the calculations, regardless of whether the due date was Saturday, Sunday or holiday. We expressed the delay of the report submitted before the due date as a negative number of days. We did not exclude extreme values to examine the timeliness based on the most available realized values. Following the above definitions, the delay always takes a smaller value than the lead time, but the IQR takes the same value regardless of the lead time and the delay. We plotted the median lead time, median delay, and IQR for each quarter in line charts.

#### Influences of changes in the operational procedures of FHSIS

Due to the differences in operational requirements of the FHSIS shown in Table 1, we divided the observation period into four: two periods and two sub-periods. The two periods was the pre-update period (1996–2008) and the post-update period (2009–2014). We divided the post-update period (2009–2014) into two: a sub-period of manual data processing (2009–2010) and a sub-period of mixed manual and computerized data processing (2011–2014). We calculated the means of the quarterly median lead times, quarterly median delays and quarterly IQRs for each of four periods.

To examine the influence of the different operational requirements on timeliness, we compared the timeliness in the four periods. First, we compared the timeliness before the update (1996–2008) with those in three periods after the update. (1) Comparison with the timeliness after the update (2009–2014) shows the overall influence of the guideline update. (2) Comparison with the timeliness in the sub-period (2009–2010) shows the influence of the extended due date and the increased number of items required. (3) Comparison with the timeliness in the sub-period (2011–2014) shows the added influence of the incomplete introduction of computerized data processing. Second, (4) we compared the timeliness between two sub-periods, which shows the influence between manual data processing, and mixed computerized and manual data processing. Third, (5) we made the above four comparisons by stratifying 22 health centers into Type 1 and Type 2.

We calculated the difference between the means of median quarterly delays and the ratio of the means of quarterly IQRs for each comparison. We performed multiple comparisons with permutation tests to check the statistical significance of the differences between the means of median quarterly delays and the ratio of the means of quarterly IQRs because the distributions of these statistics were unknown. We computed the single-step maxT adjusted p-values [12]. In the permutation test, data in each quarter of 76 quarters consisted of data from Type 1 and Type 2. As a result, the data were randomly split into a total of 152 groups according to their original sample sizes. The permutation computed the statistic from all possible permutations that constituted the distribution of the statistic under the null hypothesis [13].

The original values of the difference of the means of quarterly median delays and the ratio of the means of quarterly IQRs were used as the reference values. Out of 1000 samples, we calculated the proportion of more extreme samples than the reference values. We conducted all statistical procedures with R Ver 3.6.2 [14].

## Results

### Reporting status

**Fig 1:**
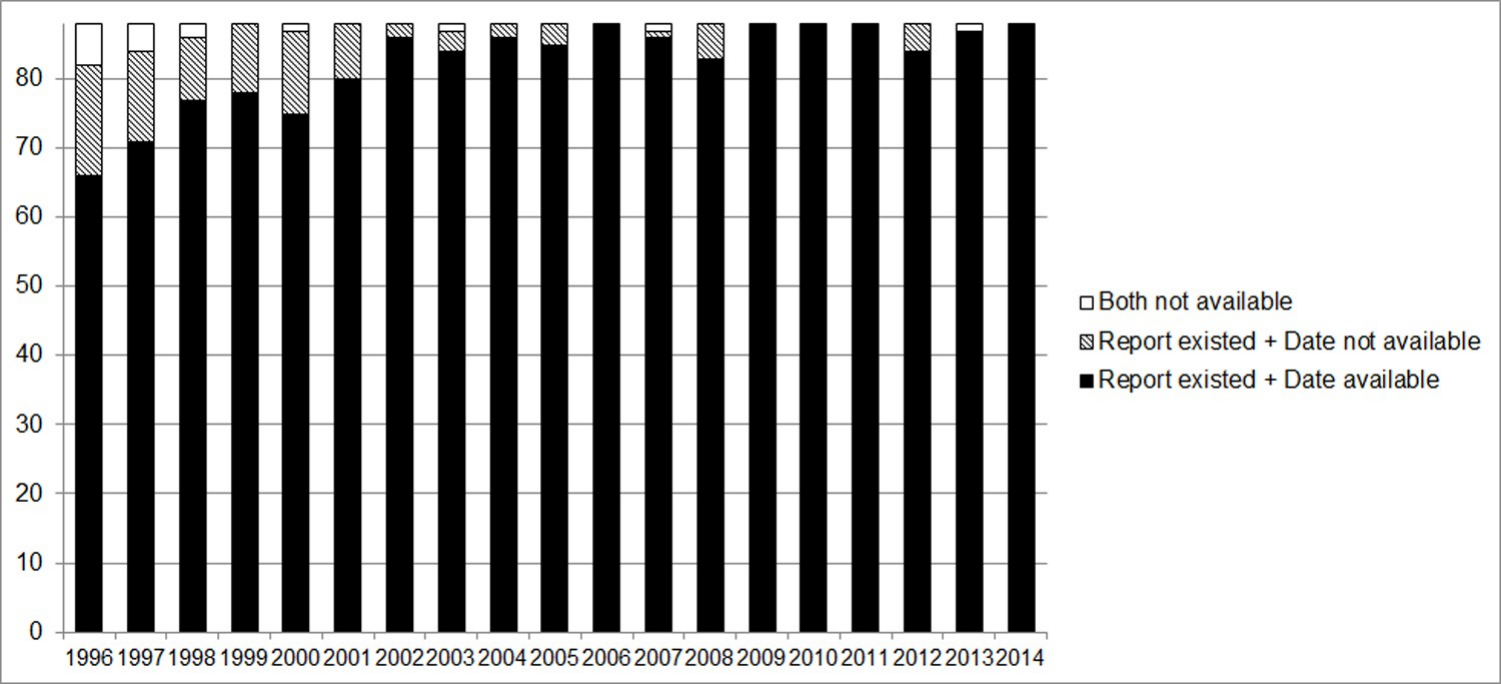
Existence of quarterly report and receipt date available. The Provincial Health Office of Palawan was supposed to annually receive 88 quarterly reports from its catchment 22 health centers. The breakdown of the stacked bar chart is the number of quarterly reports that existence was confirmed and receipt dates were available, the number of quarterly reports that existence was confirmed, but receipt dates were not available, and the number of quarterly reports that existence was not confirmed and receipt dates were not available.

The overall proportion of the quarterly reports received by the Palawan Provincial Health Office was 99.0% (1656/1672) from 1996 to 2014, while 0.96% (16/1672) of quarterly reports could not verify their existence. For 94.7% (1568/1656) of the reports received, we confirmed the date of receipt. Missing receipt dates were more common in the earlier years than in the recent years of the FHSIS operation (Fig 1).

### Timely quarterly reports

**Fig 2:**
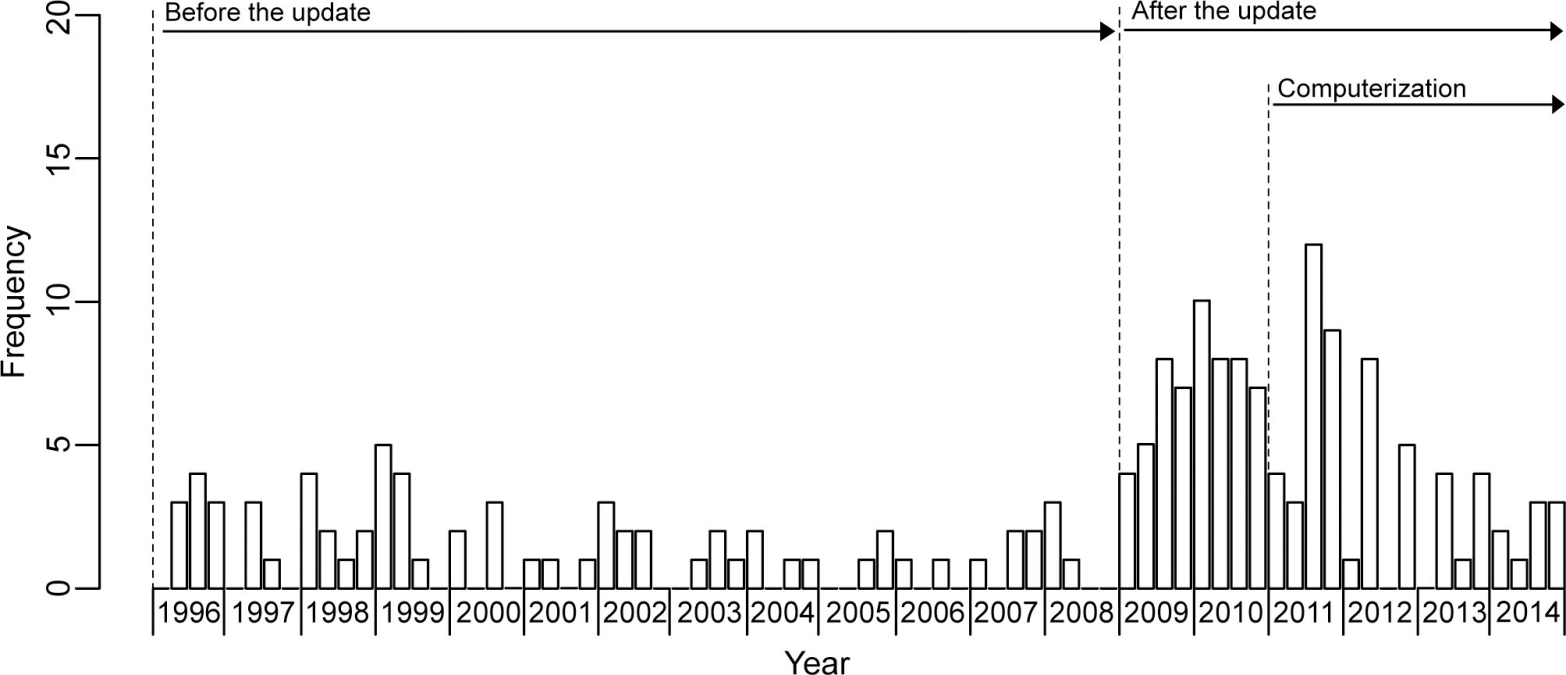
Changes in the number of timely quarterly reports. The Provincial Health Office of Palawan was supposed to quarterly receive 22 quarterly reports from its catchment 22 health centers. Operational requirements are different in the pre-update (1996–2008), the post-update (2009–2014), the sub-period (2009–2010) and the sub-period (2011–2014).

Fig 2 shows changes in the number of timely quarterly reports. The overall proportion of timely quarterly reports was 11.9% (187/1568) for the 19 years-operation. The proportion of timely reports improved from 6.7% (70/1045) to 22.4% (117/523) (OR 0.30, 95% CI: 0.22–0.41; p<0.001). During manual data processing, the proportion reached 32.4% (57/176) (OR 0.21, 95% CI: 0.14–0.31; p<0.001), and during mixed computerized and manual data processing, the proportion improved to 17.3% (60/347) (OR 0.39, 95% CI: 0.27–0.56; p<0.001). The proportion under manual data processing was significantly greater than that during mixed computer and manual data processing (OR 1.87, 95% CI: 1.24–2.82; p = 0.003).

### Influence of different operational requirements on timeliness

**Fig 3:**
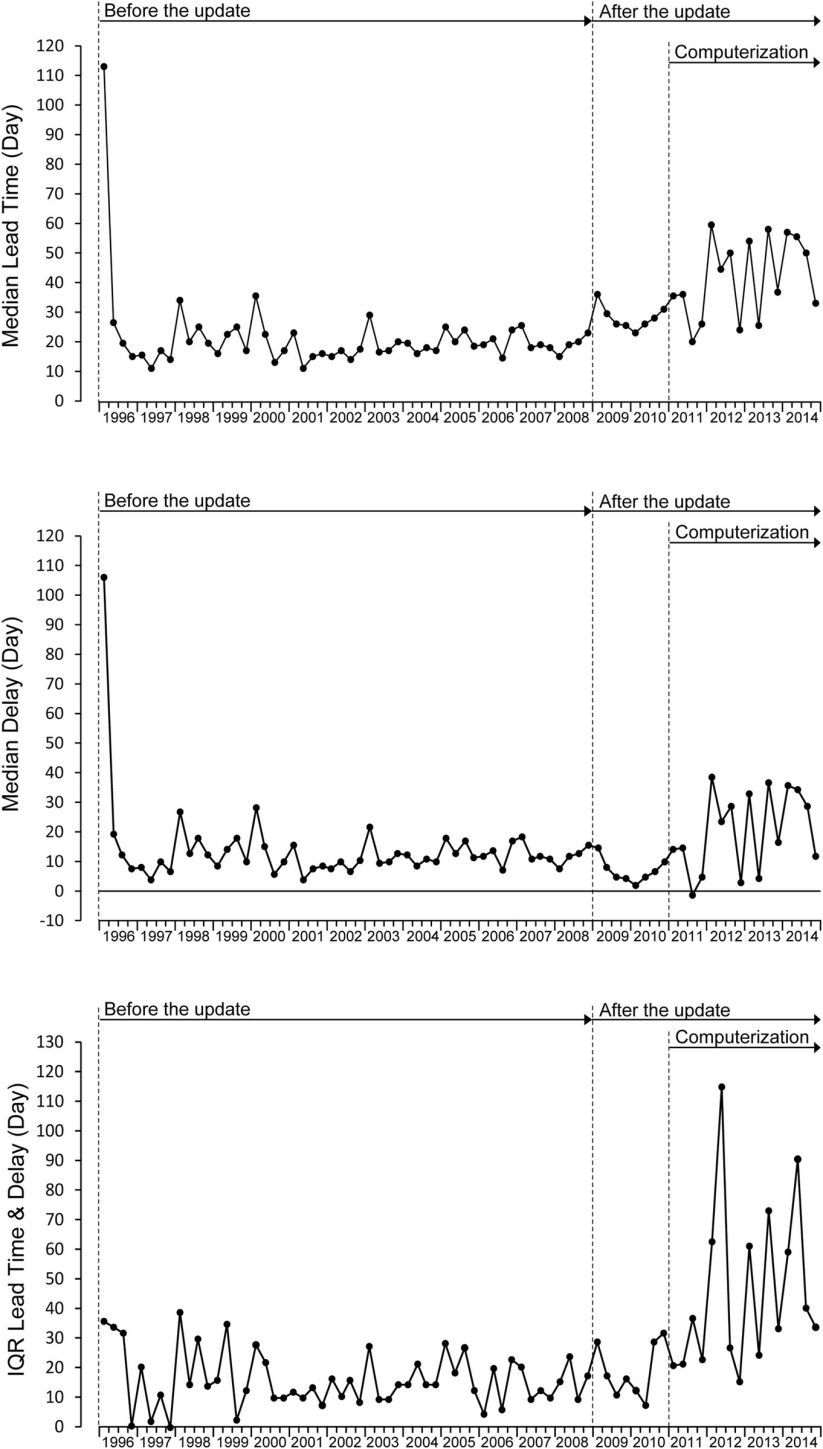
Changes in quarterly median lead time, quarterly median delay, and quarterly IQR. The upper row shows the quarterly changes in median lead time (days). The middle row shows the quarterly changes in median delay (days). The bottom row shows the quarterly changes in interquartile range (IQR).

Fig 3 shows the quarterly medians of lead time (days) and delay (days) respectively and the corresponding IQR for 22 health centers. Hereafter, “lead time” refers to the quarterly median of lead time (days), “delay” refers to the quarterly median of delay and “IQR” refers to the quarterly IQR. In the first quarter of 1996, with the introduction of the FHSIS 1996 version, we observed unusually large lead time and delay. However, the IQR was almost the same as the subsequent IQRs. It shows that the unusual lead time and delay occurred commonly in most health centers. From the second quarter of 1996 to the fourth quarter of 2008, lead times, delays, and IQRs remained stable. After the update, lead times increased due to the extension of the due date. Delays and IQRs in 2009 and 2010 were stable under manual data processing. Lead times, delays, and IQRs increased in 2011 but were stable until 2014 under mixed manual and computerized data processing.

Table 2 shows the mean of quarterly median lead times, the mean of quarterly median delays, and the mean of quarterly IQRs for four periods. Hereafter, “mean lead time” refers to the mean of the quarterly median lead times, “mean delay” refers to the mean of the quarterly median delays, and “mean IQR” refers to the average of the quarterly IQRs. The update increased the mean lead time from 21.2 days to 37.1 days. Table 3 shows the difference in mean delays and the ratio of mean IQRs for comparing the timeliness between the pre-update and three periods in the post-update. The mean delay was 14.2 days and the mean IQR was 15.7 in the pre-update. The mean delay increased by 1.9 days (p = 0.654) in the post-update, but such an increase was not significant. The mean delay decreased 7.1 days (p = 0.037) in the sub-period under manual data processing and increased 6.4 days (p = 0.037) under mixed manual and computerized data processing. The mean IQR was 2.31 times wider (p = 0.004) in the post-update. The mean IQR under manual data processing was stable at 1.19 times (p = 0.670), and the mean IQR under mixed manual and computer data processing was 2.87 times wider (p = 0).

**Table 2:** Mean lead time, mean delay and mean IQR before and after the update.

Period	Type of RHU	Mean lead time (day)			Mean delay (day)			Mean IQR
		Median	25^th^ Percentile	75^th^ Percentile	Median	25^th^ Percentile	75^th^ Percentile	
Pre-Update 1996–2008	All	21.2	14.8	30.6	14.2	7.8	23.6	15.7
	Type 1	21.9	14.8	32.3	14.9	7.8	25.3	17.6
	Type 2	20.7	13.6	29.2	13.7	6.6	22.2	15.7
Post-Update 2009–2014	All	37.1	24.2	60.6	16.1	3.2	39.6	36.4
	Type 1	45.8	30.0	73.1	24.8	9.0	52.1	43.1
	Type 2	27.4	21.2	38.4	6.4	0.2	17.4	17.2
Sub-Period 2009–2010 (Manual)	All	28.1	19.6	38.4	7.1	-1.4	17.4	18.8
	Type 1	32.3	21.5	42.9	11.3	0.5	21.9	21.4
	Type 2	25.8	18.9	30.8	4.8	-2.1	9.8	11.9
Sub-Period 2011–2014 (Mixed)	All	41.6	26.5	71.6	20.6	5.5	50.6	45.2
	Type 1	52.6	34.3	88.2	31.6	13.3	67.2	53.9
	Type 2	28.2	22.3	42.2	7.2	1.3	21.2	19.9

**Table 3:** The difference in mean delays and the ratio of mean IQRs.

Period	Type of RHU	Mean delay (days)		Mean IQR	
		Difference	p-value	Ratio	p-value
Post-Update 2009–2014	All	1.9	0.654	2.31	0.004
	Type 1	9.9	0.001	2.45	0
	Type 2	-7.3	0.022	1.10	0.864
Sub-Period 2009–2010 (Manual)	All	-7.1	0.037	1.19	0.670
	Type 1	-3.7	0.279	1.22	0.617
	Type 2	-8.9	0.005	0.76	1.000
Sub-Period 2011–2014 (Mixed)	All	6.4	0.037	2.87	0
	Type 1	16.7	0	3.10	0
	Type 2	-6.5	0.036	1.27	0.511

The mean delays and the mean IQRs in the pre-update (1996–2008) was compared with those in the post-update (2009–2014), the sub-period (2009–2010) and the sub-period (2011–2014).

### Source of increased uncertainty in timeliness

The wider mean IQR since 2011 was due to the disparity of timeliness between 15 health centers with computerized data processing (Type 1) and seven health centers with manual data processing (Type 2).

**Fig 4:**
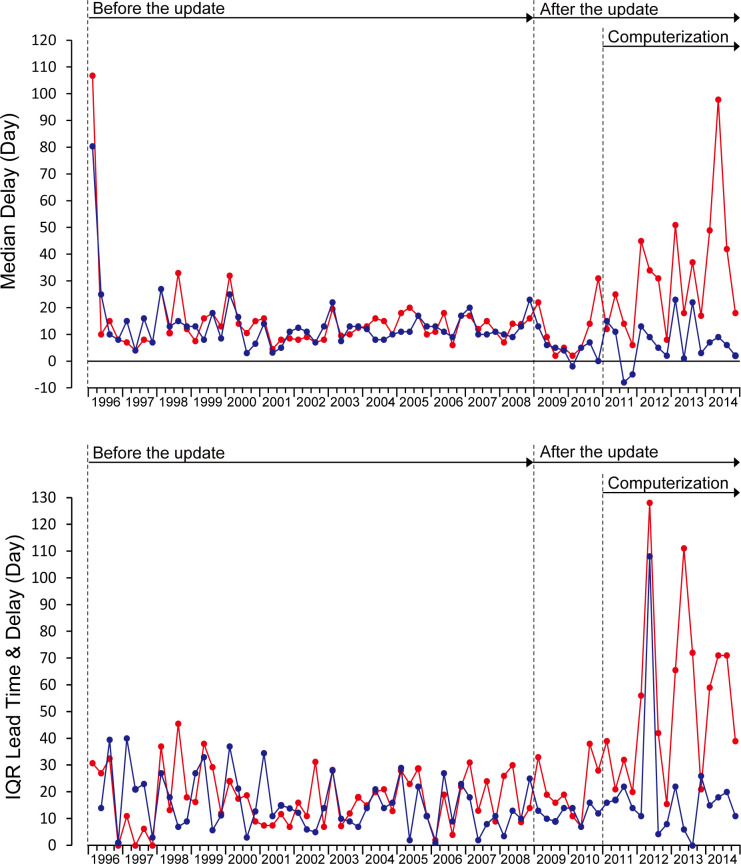
Changes in quarterly median delay and quarterly IQR in computerized data processing (Type 1) and manual data processing (Type 2). Redline: 15 health centers with computerized data processing (Type 1). Blueline: seven health centers with manual data processing (Type 2).

Fig 4 shows that after the update, both the median quarterly delay and the quarterly IQR increased in computerized data processing (Type 1). In manual data processing (Type 2), the median quarterly delay decreased, and the quarterly IQR was stable. Table 2 shows that the mean delay was 14.9 days, and the mean IQR was 17.6 before the update for the reports from 15 health centers (Type 1). Similarly, the mean delay was 13.7 days, and the mean IQR was 15.7 for the reports from seven health centers (Type 2). Table 3 shows that the mean delay increased by 9.9 days (p = 0.001), and the mean IQR increased by 2.45 times (p = 0) in Type 1 after the update. On the contrary, the mean delay decreased by 7.3 days (p = 0.022), and the mean IQR was stable at 1.10 (p = 0.864) in Type 2. Comparisons of mean delay and mean IQR between 2009–2010 and 2011–2014 showed that the mean delay increased 20.3 days (p = 0), and the mean IQR widened 2.52 times (p = 0) for Type 1. For Type 2, the mean delay increased 2.4 days (p = 0.523), and the mean IQR was 1.68 times (p = 0.068), and both were not significant.

## Discussion

The management of local health systems requires relevant indicators and quality data on health system operations. Delayed reporting hinders timely decision-making because local health systems rely heavily on RHIS for most available data. The present study found that extension of the due date increased the timely reports; however, computerization increased delays and its variation among health centers. Delay is a chain reaction. Reporting delays from health posts delay a health center’s reporting. Such delay accumulates and delays reporting to the Provincial Health Office, Regional Health Office, and the Department of Health in turn. In the Provincial Health Office. It delays the FHSIS manager’s decision to request health centers to correct data, thus delaying the revised data and provincial-wide aggregation. Therefore, timeliness needs to manage as one of the quality characteristics of RHIS.

Quality management requires controlling the level of the quality and its dispersion [15]. However, there has been little discussion on controlling timeliness in RHIS because most previous studies have observed timeliness in cross-sectional designs and do not provide sufficient information to discuss the certainty of the timeliness.

By observation of 76 quarters, the present study found that (1) the proportion of received reports was higher if we observed them beyond the due date, taking into account delays in submission; (2) lead time and delay tended to converge to a stable state, even if the stable state did not necessarily meet the due date requirement; (3) interventions for controlling timeliness were effective when they had continuity with the previous operational procedures. Extension of the due date increased the timely reports. Abrupt changes in operational procedures, such as computerized data processing, caused significant delay and uncertainty. These findings highlight opportunities for improvement in both the design and operation of RHIS.

### High proportion of reports submitted to RHIS

In Mozambique, the high proportion of report submissions was confirmed by observing the submission status of monthly reports over 12 months [16]. Similarly, this study confirmed the high proportion of submission of quarterly reports in Palawan province by observing the submission status over 76 quarters. Since reports are sometimes submitted late, both studies observed a certain period regardless of the due date. For example, it took two quarters to obtain the most delayed report in this study. It suggests that the proportion of reports submitted to the RHIS would be higher than previously known results if there were a sufficient observation period to capture the delayed reports. Given that the submission of reports by civil servants is an obligation given by national authority, the decision to submit or not to submit a report can make a big difference for RHIS managers. Even if it is difficult for the RHIS manager to prepare the report, not submitting the report would be the last option unless there is a reasonable reason.

### Systematic delay, attained timeliness and its stability

Delay in reporting depends on the due date. It is known that teams are more likely to complete their tasks in time if there is shared temporal cognition among team members [17]. In Palawan, all the health centers had a temporal cognition of reporting by the given due date of the FHSIS. The Provincial Health Office of Palawan had repeatedly reminded the health centers of the due date. In this study, few reports met the expected timeliness although the lead time was in a stable state in the pre-update. If the expected timeliness is too high for the achievable lead time, the observed delays can be attributed to a system design that requires too high expectations [18].

The national level develops RHIS standards to realize the intended performance and quality of RHIS. The local level then adapts its routines to the given standard. In this sense, the lead time obtained in this study represents the local specification of the FHSIS.

Setting achievable goals will help gradually achieve the given standards. In practice, even if the obtained lead time did not meet the expectation of the FHSIS, FHSIS managers will set more realistic due dates for their respective locality. In this case, controlling a usual state of performance is essential to reach a better level of performance in the future [19]. In the present study, we found the stable timeliness in the pre-update continued for eight quarters in the post-update under manual data processing. Also, a usual state of performance helps identify an occurrence of an unusual state like seen in the first quarter in 1996. The obtained timeliness needs to be tracked over time in RHIS operation to use the benefit of a usual state of timeliness.

### Improvement of timeliness by the design of FHSIS

The update increased timely reports and sustained delay and IQR under manual data processing even though the burden of data processing increased. However, when 15 health centers introduced computerized data processing, the proportion of timely reports returned lower, like in the pre-update. The increase of timely reports was probably because health workers coped with the increased burden with familiar procedures in enough time. However, computerized data processing would have been unfamiliar tasks that brought a significant and radical change in the routine of 15 health centers. If computer literacy is necessary to adapt to the computerized procedures, the design of the RHIS must consider necessary resources for all health centers in the Philippines to adapt computerized data processing.

Our finding suggests that the key to an effective intervention is to introduce modest operating procedure changes that extend the existing procedures to which the managers and staff responsible for data processing has already empirically accustomed. This lesson would benefit RHISs with limited resources available for frequent and ongoing training on new operating procedures.

### Improvement through innovation

Improvement by innovation such as the introduction of Information Communication Technology (ICT) can rapidly improve the performance and quality of RHIS. In Uganda, the computerized RHIS called DHIS2 has improved report submission status and timeliness of report submission [20]. It was hoped that the successful introduction of ICTs would also benefit the FHSIS in the Philippines. However, in the early stage of ICT implementation in the FHSIS, this study found that deterioration of timeliness was due to the digital divide [21] among health centers. Seven health centers with manual data processing (Type2) had better timeliness than those with 15 computerized health centers (Type 1). Manual data processing was unavoidable for five out of the seven health centers (Type 2) located on islands, where power outages could often interrupt a computer.

For 15 health centers (Type 1), computerization added a new task in the data encoding process: typing the data into a computer. This task seems easy for the younger generation, but it must have been unfamiliar for health workers of a certain age who have never touched a computer before. The computerized data consolidation process would have the advantage of automatic consolidation if the data was encoded into a computer program. However, the initial eFHSIS program had problems with not all the health posts in each municipality reflected in the list, thus incorrect aggregation results. Therefore, in some cases, manual consolidation was done in parallel to verify the consolidated data by eFHSIS. The internet environment was unlikely to affect timeliness because only seven out of 22 health centers used online reporting. Five out of seven health centers (Type 2) did not have the internet in island areas. Eight out of 15 municipalities (Type 1) had poor connections even with an internet environment. Those health centers had to save the report data on storage media or submit paper-based reports. In addition, the text was too small to be read when the aggregated results were reflected in the report forms by the eFHSIS program. Unfamiliarity with the new tasks and the initial bugs in a computer program could attribute to the loss of timeliness despite the computerization.

On the contrary, the health centers that continued manual data processing were able to handle data as an extension of the existing routines and thus cope with the increased demands of the RHIS.

New technologies have triggered changes in organizational routines, but solving problems such as the digital divide needs to be discussed at the locality and the national level. RHIS designers must minimize problems caused by the design of the RHIS itself, especially when new technologies add unfamiliar tasks to health workers.

### Limitations

Certain limitations of the study should be borne in mind. First, the findings of this study may not be fully applicable to other areas in the Philippines. Data were collected only from health centers in 22 municipalities under the Provincial Health Office of Palawan. Since Palawan is the largest province in the Philippines, consisting of about 1,780 islands and islets, these health centers may be more difficult to access to the Provincial Health Office. In addition, the results of the permutation test do not assure external validity. Second, our inference is based on the available samples of 94.7% (1568/1656) of reports confirmed during the 19 years of the FHSIS operation. The remaining 0.96% (16/1672) of reports may be submitted but missed during the operation of the FHSIS. If this is the case, the timeliness of reports, especially in the earlier years, may have been biased by these missing reports. Third, a psychological influence on an official setting of the due date was beyond the scope of this study. Procrastinators may partially cause more extended preparation periods. Finally, the relationship between the timeliness, the accuracy and the completeness of the data remains unclear. In theory, trade-offs between multiple quality characteristics need to be considered to set feasible goals. In practice, however, the performance and quality of data timeliness are often readily recognized in the workplace. Improved data timeliness also allows RHIS managers to check data for completeness and accuracy more quickly and encourage health centers to correct the data sets when identified as necessary.

## Conclusion

Time-series observation of the timeliness of the FHSIS provided gauges of usual and unusual timeliness and dispersion around the timeliness. Extending the due date increased timely reporting. However, introducing unfamiliar tasks increased delays and uncertainty in timeliness. In a low-resource setting, effective interventions in RHIS need to consider modest changes of operating procedures that extend the existing routines the staff in charge has already accustomed to. More attention must be paid to controlling uncertainty in RHIS.
